# Building laboratory capacity to detect and characterize pathogens of public and global health security concern in Kenya

**DOI:** 10.1186/s12889-019-6770-9

**Published:** 2019-05-10

**Authors:** Elizabeth Hunsperger, Bonventure Juma, Clayton Onyango, John B. Ochieng, Victor Omballa, Barry S. Fields, M. Kariuki Njenga, Jane Mwangi, Godfrey Bigogo, Richard Omore, Nancy Otieno, Sandra S. Chaves, Peninah Munyua, Daniel Macharia Njau, Jennifer Verani, Sara Lowther, Robert F. Breiman, Joel M Montgomery, Kevin M. De Cock, Marc-Alain Widdowson, CDC and KEMRI Laboratory and Epidemiology Team, Evans Apondi, Jeremiah Nyaundi, Lillian Mayieka, Lydia Mwasi, Newton Wamola, Stella Gikunju, Gilbert Kikwai, Caroline Ochieng, Timothy Mujete, Dennis Odhiambo, Melvin Ochieng Ogolla, Everlyne Abisinwa, Robert Mugoh, Herrine Odiembo, Frederick Ade, Jane Juma Onyiengo, Jim Katieno, Patrick Emojong, Shirley Lidechi, Baryl Achieng, Arthur Okloyo, Dorothy Odindo, Wilfrida Agai, Carol Oumo, Lillian Arita, Jairus Abuom, Derrick Amon, Loicer Achieng, Mike Osita, Mose Alando, Josh Mott, Henry Njenga, Linus Ndegwa, Rachael Joseph, Maurice Ope, Charles Okello, Cyrus Wachira, Kabura Wamburu, Lilian Wakhule, Carolyne Jeruto, Solomon Gikundi, Leonard Nderitu, Sylvia Omulo, Rose Wanjala, Joshua Obiya, Patricia Ngotho, Regina Ngore, George Awiti, Wilson Gumbi, Kipyegon Korir, Collins Oungo

**Affiliations:** 1 Centers for Disease Control and Prevention (CDC), Center for Global Health (CGH), Division of Global Health Protection (DGHP), Nairobi, Kenya; 2 CDC, CGH, DGHP, Epidemiology, Informatics, Surveillance and Laboratory Branch, Atlanta, GA USA; 3 Kenya Medical Research Institute (KEMRI), Center for Global Health Research (CGHR), Kisumu, Kenya; 4 KEMRI, CGHR, Nairobi, Kenya; 5 Washington State University, Pullman, WA USA; 6 CDC, National Center for Immunization and Respiratory Diseases, Influenza Division, Nairobi, Kenya; 7 CDC, DGHP, Workforce Institute Development Branch, Nairobi, Kenya; 8 CDC, CGH, Division of Global HIV and TB (DGHT), Nairobi, Kenya; 9 Emory Global Health Institute, Emory University, Atlanta, GA USA

**Keywords:** Diagnostics, TaqMan Array card (TAC), Laboratory capacity, Biosafety, Global health security

## Abstract

Since 1979, multiple CDC Kenya programs have supported the development of diagnostic expertise and laboratory capacity in Kenya. In 2004, CDC’s Global Disease Detection (GDD) program within the Division of Global Health Protection in Kenya (DGHP-Kenya) initiated close collaboration with Kenya Medical Research Institute (KEMRI) and developed a laboratory partnership called the Diagnostic and Laboratory Systems Program (DLSP). DLSP built onto previous efforts by malaria, human immunodeficiency virus (HIV) and tuberculosis (TB) programs and supported the expansion of the diagnostic expertise and capacity in KEMRI and the Ministry of Health. First, DLSP developed laboratory capacity for surveillance of diarrheal, respiratory, zoonotic and febrile illnesses to understand the etiology burden of these common illnesses and support evidenced-based decisions on vaccine introductions and recommendations in Kenya. Second, we have evaluated and implemented new diagnostic technologies such as TaqMan Array Cards (TAC) to detect emerging or reemerging pathogens and have recently added a next generation sequencer (NGS). Third, DLSP provided rapid laboratory diagnostic support for outbreak investigation to Kenya and regional countries. Fourth, DLSP has been assisting the Kenya National Public Health laboratory-National Influenza Center and microbiology reference laboratory to obtain World Health Organization (WHO) certification and ISO15189 accreditation respectively. Fifth, we have supported biosafety and biosecurity curriculum development to help Kenyan laboratories safely and appropriately manage infectious pathogens. These achievements, highlight how in collaboration with existing CDC programs working on HIV, tuberculosis and malaria, the Global Health Security Agenda can have significantly improve public health in Kenya and the region. Moreover, Kenya provides an example as to how laboratory science can help countries detect and control of infectious disease outbreaks and other public health threats more rapidly, thus enhancing global health security.

## Background

Since 1979, the US Centers for Disease Control and Prevention (CDC) has worked in collaboration with the Kenya Medical Research Institute (KEMRI), Ministry of Health (MoH) and other key government institutions as partners to conduct public health science of national, regional and global relevance. Initially this work focused on malaria, tuberculosis (TB) and human immunodeficiency virus (HIV) and these efforts established the groundwork of this laboratory collaboration in Kenya. Then in 2004, the Kenya CDC Global Disease Detection (GDD) program within the now-named Division of Global Health Protection (DGHP) was established along with 10 other sites globally following the international outbreak of Severe Acute Respiratory Syndrome (SARS), and subsequent expansion of Influenza A/H5N1 globally. Later in 2006, the CDC Kenya Influenza Program was established, to provide specific focus on influenza surveillance, epidemiology, control and prevention. As part of the collaboration between KEMRI and DGHP, the Diagnostics and Laboratory Systems Program (DLSP) was developed comprising KEMRI and DGHP-Kenya scientific laboratory technical staff, aimed to support the establishment of diagnostic laboratory capacity at KEMRI for surveillance, epidemiological studies, outbreak investigations, vaccine trials, and clinical research. In parallel, complementary laboratory programs in the CDC Divisions of Global HIV and TB (DGHT), operating with funds from the President’s Emergency Plan for AIDS Relief (PEPFAR), and the Division of Parasitic Diseases and Malaria (DPDM) support similar work targeting those specific diseases. In 2014, concurrent with the West Africa Ebola epidemic, the Global Health Security Agenda (GHSA) was initiated, providing a framework to expand DSLP laboratory capacity building efforts.

DSLP currently has several objectives in Kenya. First, to support infectious disease surveillance for acute respiratory infections, diarrheal diseases, vector-borne, viral hemorrhagic fever and zoonotic diseases in order to detect pathogens, determine disease burden and the impact of intervention studies. Second, to support outbreak response in order to detect epidemic pathogens and allow for rapid implementation of pathogen-specific control measures. Third, for diagnostic development and evaluation to allow for advanced pathogen characterization and discovery. Fourth, the development of laboratory capacity for the Government of Kenya (GoK) and the East African region in alignment with the International Health Regulations (IHR) with the expectation that all countries have the capacity to rapidly detect and control infectious disease outbreaks and other public health threats at their source. Lastly is the implementation of biosafety and biosecurity capacity in Kenya. This manuscript outlines the achievements of DLSP over the years in achieving these core objectives.

### Overview of DSLP laboratories

**Fig. 1:**
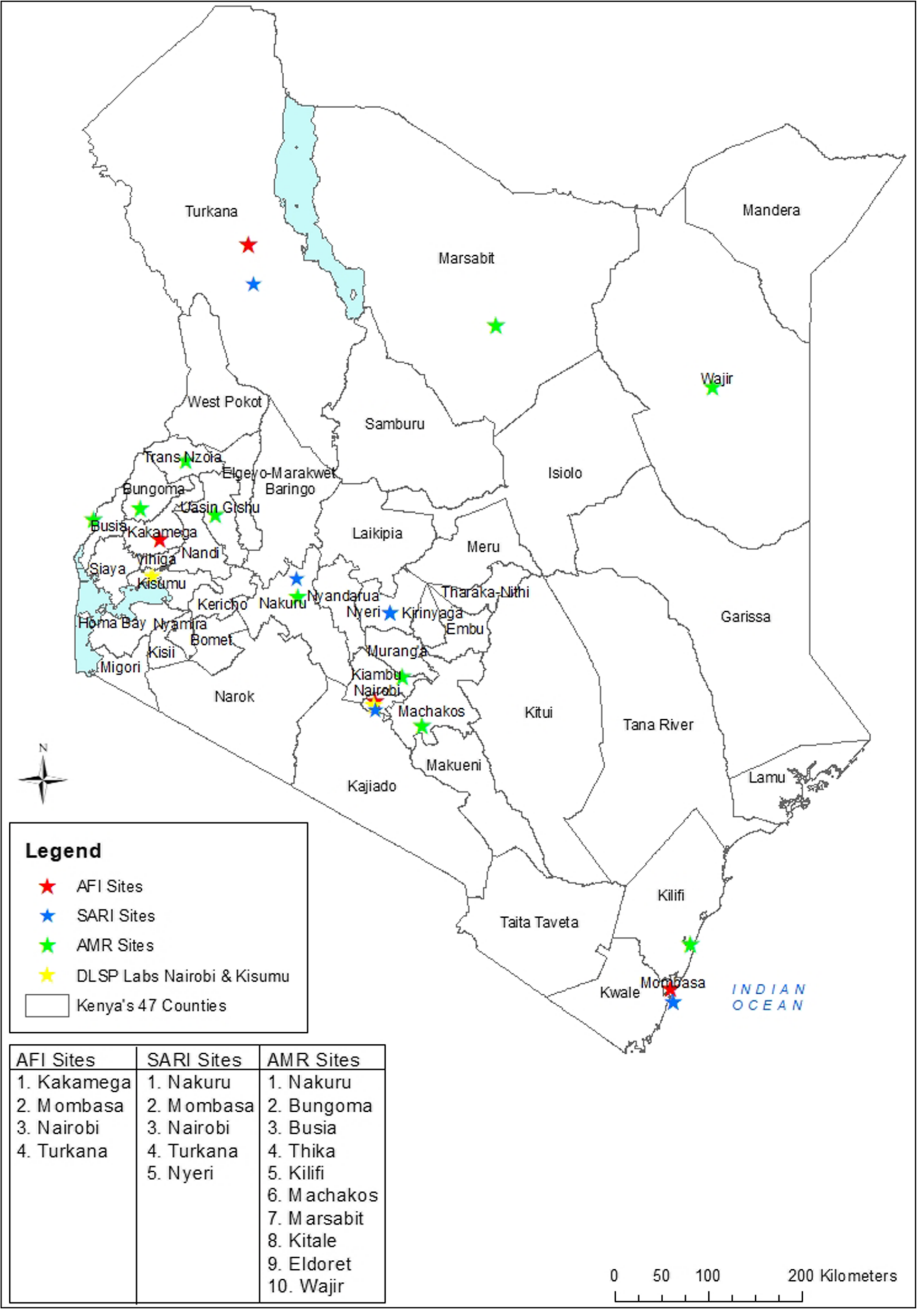
Physical location of Diagnostics and Laboratory Systems Program (DLSP) in Kenya and 10 county laboratory systems strengthening for the detection of Antimicrobial Resistance (AMR), 4 Acute Febrile Illness (AFI) surveillance sites and 5 National Influenza Surveillance sites

DLSP current activities take place in three KEMRI laboratories, one in Kisumu and two in Nairobi (Fig. 1). The KEMRI Kisumu facility houses zoonotic, biological safety level 3 (BSL3), respiratory and enteric laboratories. The Nairobi KEMRI facility has a BSL3 laboratory where Taqman Array Card (TAC), Next Generation Sequencing (NGS) and cell culture are performed. The KEMRI Kisumu and Nairobi laboratories also include HIV, malaria and TB laboratories. A third satellite microbiology laboratory is located in Kibera, an informal urban settlement in Nairobi. This microbiology laboratory supports population-based surveillance at the Tabitha Clinic, operated jointly with Carolina for Kibera, a non-governmental organization (NGO) providing health and other services for the people of Kibera.

### Determining burden of disease and monitoring disease interventions

Laboratory-based surveillance is critical in accurately assessing trends in the incidence and prevalence of a disease and for monitoring impact on morbidity or mortality of disease-or-pathogen targeted interventions. DSLP supports several ongoing surveillance platforms for enteric and respiratory disease, acute febrile illness (AFI) and jaundice. Disease burden and intervention effectiveness cannot be easily translated from one setting to another, especially when the epidemiology, circulating strains or co-morbidities and other ecologic considerations are dissimilar. To introduce and sustain prevention programs such as vaccination, policy-makers need evidence of disease burden and impact of the intervention among other factors. DLSP has contributed to national and global burden estimates for a range of pathogens, most notably enteric and respiratory pathogens [1–10] These estimates have led to the introduction of vaccines against rotavirus (in 2014) and pneumococcus (in 2011) in Kenya. Diagnostic testing is key in these studies- an example is rotavirus vaccine efficacy which varies significantly between high and low income settings [11] may be due to the different genotypes circulating than those targeted in the vaccines, poor nutrition or environmental enteropathy or other factors such as competition with co-existing enteric pathogens [12].

Between 2014 and 2017, DSLP provided laboratory support for two observational rotavirus vaccine studies: Rotavirus-Vaccine Impact on Diarrhea in Africa (R-VIDA), a multi-country study that includes Kenya, Mali and Gambia; and Rotavirus Immunization Program Evaluation in Kenya ((RIPEK), a multi-site study within Kenya) to determine the effectiveness of the rotavirus vaccine. Recent influenza studies in Kenya that were supported by DLSP assisted in describing influenza disease burden in the country which was crucial information used by the Kenyan National Immunization Technical Advisory Group (KENITAG) for the recommendation of influenza vaccination for children ages 6–23 months [13–15]. In addition, this diagnostic capacity enabled other complex clinical research projects including Pediatric Respiratory Etiology Surveillance Study (PRESS) and Child Health and Mortality Prevention Surveillance (CHAMPS) that collect data to describe the causes of under-five child mortality in high-mortality settings [16]. The CHAMPS study is a network study involving several countries and has a broader approach by describing under-five child mortality from both sentinel health facilities and the community. This project collects tissue specimens and blood to evaluate multiple pathogens using TAC and histopathology. The PRESS project aimed to specifically evaluate respiratory pathogens associated with mortality among children under-five years and compared diagnosis by TAC with histopathology techniques in a major hospital in Nairobi.

Other zoonotic studies that DLSP has supported include those on brucellosis in humans and animals and risk factors for brucellosis transmission such as ingestion of raw milk, exposure to goats, and handling of animal hides, as well as the risk of transmission of other enteric pathogens from animals to humans [17, 18]. DLSP has also evaluated serologic evidence of human exposure to *Bacillus anthracis*, *Brucella* spp., spotted fever group rickettsioses, and typhus group rickettsioses from persons aged 15–64 years and determined a high seropositivity suggesting frequent exposure to these pathogens in the Kenyan population. These studies allowed a recent prioritization of zoonotic diseases of public health importance in Kenya [19, 20].

### Outbreak investigations

**Fig. 2:**
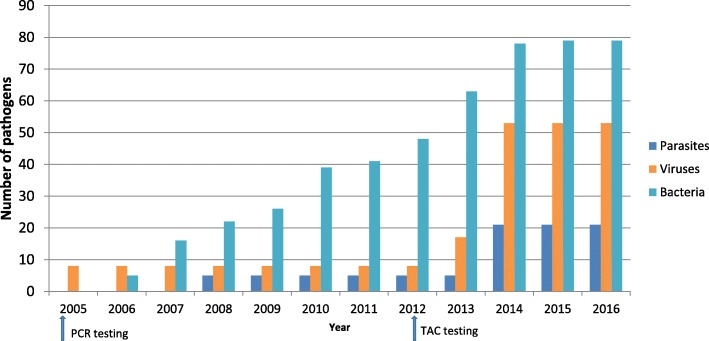
Number of parasites, viruses and bacterium in human, animal and environmental specimens the DLSP laboratory can test using diagnostic assays by year. Blue = parasites, orange = viruses, teal = bacterium. Arrows represent implementation of Polymerase Chain Reaction (PCR) and TaqMan Array Card (TAC)

**Fig. 3:**
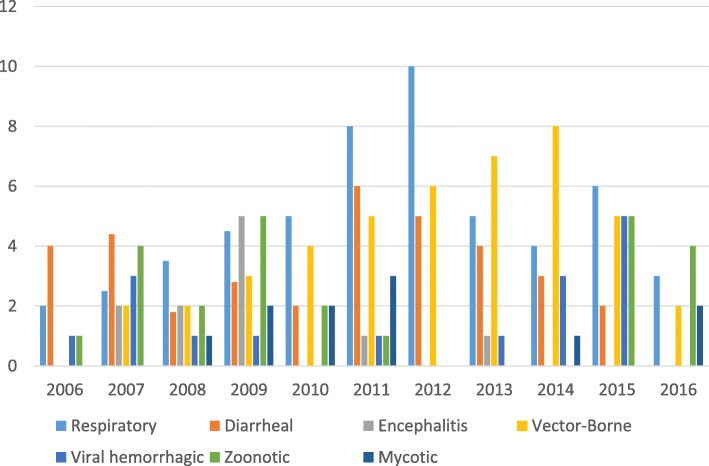
Number of annual outbreak responses in Kenya and the region by disease syndrome supported by DLSP

**Fig. 4:**
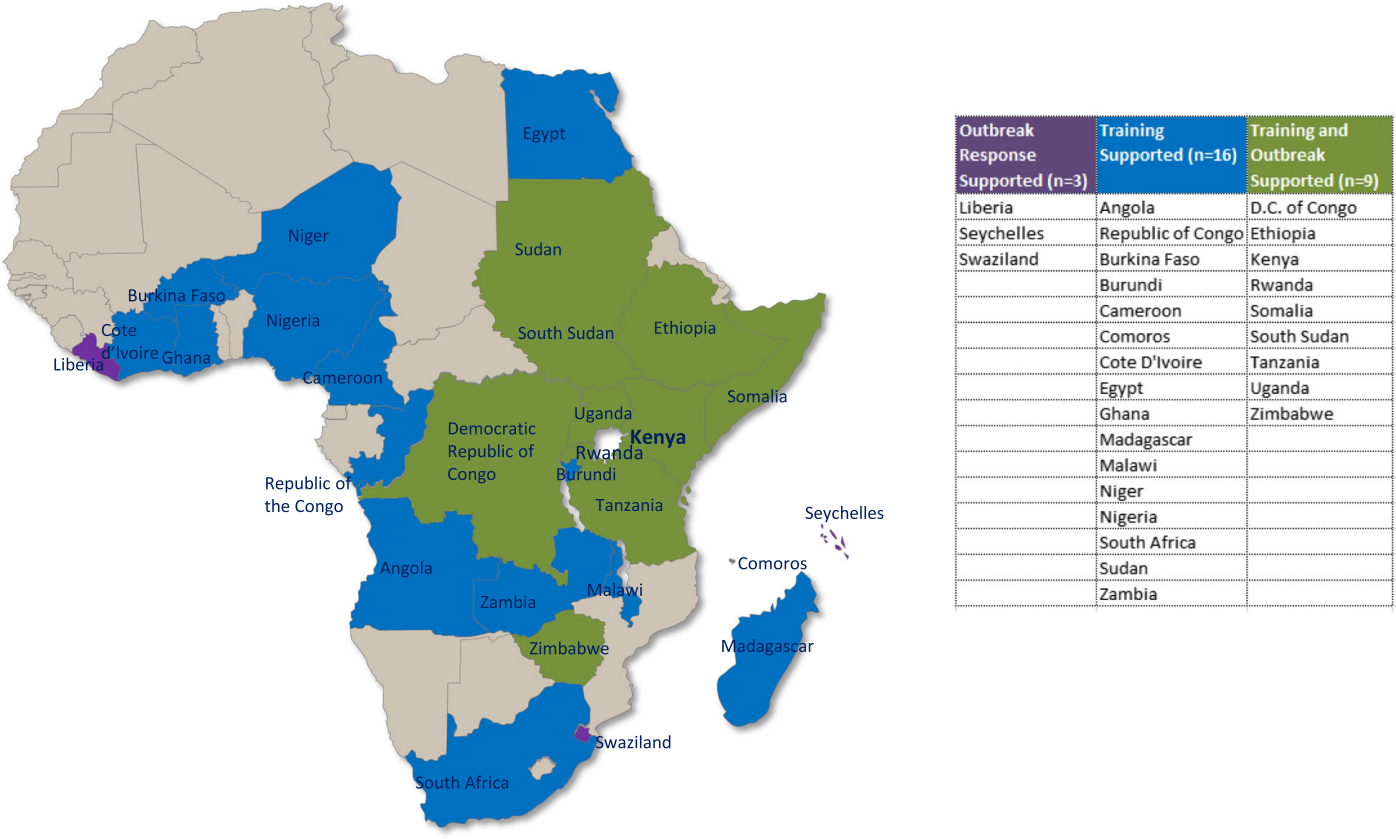
Countries that DGHP supported for training and/or outbreak response from 2006 to 2016

DLSP has had a central role as a reference laboratory in Kenya and the African region because of its ability to detect many pathogens with a variety of techniques (Figs. 2 and 3b). DSLP advanced laboratory capacity complements the current NPHLS testing capacity as a reference laboratory during outbreak responses for the Kenya MoH. Moreover, each year over the last 10 years, DGHP expertise both in epidemiology and laboratory has provided support for multiple outbreaks investigations in Kenya and in Africa (Figs. 3 and 4) [21]. In Kenya, DGHP has supported investigations into outbreaks of respiratory diseases (e.g., influenza, pertussis), diarrheal diseases (e.g.*,* cholera, *Salmonella* Typhi, shigellosis), aflatoxicosis, and vector-borne diseases (e.g., Zika, dengue, chikungunya, Rift Valley fever viruses) (Fig. 3) [9, 22–27]. These outbreak investigations are in support of the MoH outbreak investigations conducted by the Disease Surveillance and Response Unit and Field Epidemiology and Laboratory Training Program (FELTP) or organizations such as United Nations High Commissioner for Refugee and NGOs, dealing with outbreaks in refugee camps. The Kenya FELTP program has partnered with DLSP in these outbreak investigations and has played a central role. Kenya FELTP was modeled after the 60-year-old CDC Epidemic Intelligence Service, which trains field epidemiologists to operate public health surveillance and response systems in the U.S. The Kenya FELTP has enrolled and developed laboratory scientists, physicians, and other health scientists as epidemiologists to operate public health laboratories and networks, public health surveillance and response systems since 2004.

For DGHP Africa regional Ebola outbreak response activity in 2014, a joint CDC Kenya DLSP staff and US National Institutes of Health team was deployed to Liberia, where the first mobile laboratory during the Ebola outbreak was established that assisted in the real time reverse transcription polymerase chain reaction (rRT-PCR) confirmation of Ebola virus from suspected and probable cases of Ebola virus disease for clinical management. The mobile Ebola laboratory tested ~ 6000 specimens in support of the Ebola response [28]. DLSP has also identified and responded to other outbreaks including a rickettsial outbreak in Rwanda, chikungunya in Comoros, Rift Valley Fever (RVF) in Tanzania, Hepatitis E in South Sudan, and sandfly fever Sicilian virus in Ethiopia [29]. The complete number of outbreaks and training performed in the region is summarized in Fig. 4.

### Development of new diagnostic technology to detect emerging and re-emerging pathogens

The diagnostic capacity in DLSP laboratories has been augmenting annually since 2004. To date DLSP, across its three participating laboratories, is capable of testing for 153 different pathogens by rRT-PCR, serological assays, microscopy, bacterial and viral culture, immunofluorescence and TAC. (Fig. 2). DLSP has been a leader in the development, validation and field evaluation of TAC technology capable of detecting > 25 pathogens from a single specimen from cases of common syndromes AFI, respiratory disease, enteric disease, encephalitis). TAC is a microfluidic PCR array platform that allows the customization of pathogen targets according to the needs of each study. Four main TACs were developed, evaluated and/or validated by DLSP (AFI, Enteric, Encephalitic and Respiratory). The AFI TAC has been validated in Kenya and other GDD centers and used throughout the GDD network of laboratories [30]. This TAC targets 25 pathogens (14 viral, 8 bacterial and 3 protozoa) with an overall sensitivity of 88% and specificity of 99% compared to conventional RT-PCR [30]. The Enteric TAC was similarly validated for 15 of the most common enteropathogens detected in the Global Enteric Multicenter Study (GEMS) using 1500 clinical specimens [31, 32]. The Encephalitis TAC was developed to detect 21 pathogens (2 parasites, 6 bacteria, 13 viruses) known to cause encephalitis, with an overall sensitivity of 86% and specificity of 97% across tested comparative assays [33]. Because TAC is an effective screening tool for multiple pathogens in a single sample, it can detect co-infections or discover emerging pathogens not routinely tested through national surveillance platforms that frequently have a narrow focus.

As part of the DLSP collaboration, DGHP has recently acquired next generation sequencing (NGS) technology in KEMRI laboratories. NGS technology has expanded beyond the 1970s Sanger technology and 1988 capillary electrophoresis based sequencing instruments allowing for high throughput to produce 1.8 terabases in a single run which is approximately 1000-fold increase from the first generation sequencing technology. NGS is also capable of identifying new and multiple pathogens from single specimens [34] as well as further characterizing existing pathogens for phylogenetic analysis using metagenomics to detect microbiomes from different specimens (e.g.*,* stool, blood, skin) [35]. The main advantage of whole genome (WG)-NGS is that there is no need to design primers by pathogen target and the laborious process of testing primers for specificity and assessment of their interference with other targets and will likely replace PCR technology as routine diagnostic test. WG-NGS has successfully identified a new rickettsial species, *Candidatus Rickettsia asembonensis* strain NMRCiiT [36]. Sequencing studies for use in characterizing the spread of influenza viruses, cholera, and vector-borne pathogens such as chikungunya, dengue and Zika viruses are in progress. Overall DLSP has participated in over 50 research projects in Kenya and scientists within DLSP were co-authors of over 180 peer-reviewed published articles.

### Laboratory systems and capacity building

Since 2014, DSLP has joined with other CDC laboratory programs to further build national laboratory capacities, and augment previously established laboratory strengthening efforts. We began by identifying three important gaps within the national system [37]. First, we identified gaps at the national level and augmented the capacity of NPHLS for both reference bacteriology (including antimicrobial resistance [AMR] testing) and influenza diagnostics in order to support the county laboratories. Second, we identified the gaps at county level by mapping the tiers 2 through 6 national laboratories within Kenyan county hospitals. Concurrent with these activities, DLSP strengthened the laboratory information system (LIS), by building new capacity on an existing platform developed by DGHT at the national and county levels to increase and improve pathogen reporting for surveillance purposes. Lastly, we identified gaps in specific diagnostic capacities and DSLP contributed to Kenyan work force development in the form of specific laboratory training for instance on AMR testing and hepatitis diagnosis coupled with mentoring of laboratory technical staff for microbiology and molecular biology techniques.

NPHLS is the national reference laboratory for Kenya MoH, that serves to confirm or test specimens submitted from all other tier level county hospital laboratories within the network. Two important gaps within NPHLS was the capacity of both the microbiology and influenza laboratories. In July 2016, DGHP, with support from the East African Public Health Laboratory Network (EAPHL), the World Bank, the American Society for Microbiology and FIND, assisted in preparing the MoH-NPHLS Microbiology reference laboratory for ISO15189 accreditation. There were 8 key elements needed for accreditation: 1) developing Standard Operating Procedures for all microbiology tests used for clinical diagnosis, 2) laboratory training for all microbiology assays 3) training for microbiology equipment (e.g., BACTEC), 4) equipment preventive maintenance, 5) participation in External Quality Assessment panel for microbiology 6) internal quality control 7) quality improvement protocols and 8) process improvement and control protocols. To ensure a successful ISO 15189 accreditation, DGHP worked together with MoH on all non-conformities identified by WHO auditors. Similarly DGHP assisted and prepared KEMRI laboratories operating as DLSP to successfully obtain ISO 15189 accreditation for its Nairobi and Kisumu laboratories in 2014.

WHO accreditation of NPHLS for microbiology provided a framework for the implementation of AMR surveillance and strengthening of county laboratory capacity initiated through a pilot study in two county referral hospitals (Thika and Kitale counties) where DLSP trained county staff in stockpile management, sample collection, sample packaging, shipment and specimen chain-of-custody to support microbiology laboratories in these regions. Establishing an effective system for AMR characterization and surveillance requires a properly functioning microbiology laboratory with inventory management, quality media production, biochemical testing, serotyping, drug susceptibility testing capacity, specimen referral system, and shipping. In preparation for AMR testing, DGHP with support of the Defense Threat Reduction Agency (DTRA) and DGHT, assisted in developing the KEMRI Production Unit for the manufacturing of prepared microbiology reagents (e.g.*,* MacConkey agar, chocolate agar and sheep blood agar plates) to facilitate microbiology testing at both NPHLS and tier 2–6 hospital laboratories throughout Kenya. DLSP, in collaboration with NPHLS, provided training for microbiology in the AMR pilot sites at Thika and Kitale as well as mentorship in order to systematically implement this capacity throughout Kenya. Within 3 years, the project will expand to include an additional 8 counties with improved capacity in microbiology techniques, AMR, specimen referral and shipping (Fig. 1).

Another GHSA laboratory capacity building activity implemented by DLSP is the transfer of the diagnostic capability of the National Influenza Center (NIC) from a CDC/KEMRI program, to the GoK MoH-NPHLS. DGHP and the CDC Influenza Program provided state-of-the-art molecular equipment and training to MoH-NPHLS technical staff to successfully diagnose influenza viruses. The capacity for rRT-PCR used for influenza detection will also be leveraged for the detection of other viral respiratory pathogens of public health interest such as Respiratory Syncytial Virus, Middle East Respiratory Syndrome Coronavirus (MERS-CoV) and other emerging or reemerging respiratory infections. This enhanced surveillance effort is part of an initiative called Detection and Response to Respiratory Events which will incorporate both viral and bacterial respiratory pathogens. As of 2016, NPHLS NIC has successfully assumed diagnostic testing responsibility for 2 of the 5 influenza sentinel surveillance sites in the country (Fig. 1). However, in order for the NIC to report surveillance data to the WHO network, NPHLS is required to be a certified WHO-NIC – the final step is the finalization of virus culture capacity expected to be completed by 2018.

In order to identify county gaps, DSLP supported a laboratory mapping exercise to describe the diagnostic capacities of all hospital GoK laboratories designated as tier 2 through 6 within the 47 counties of Kenya and their referral and reporting systems and patterns by tier status. The Kenyan laboratory network is a decentralized system where the counties operate independently from NPHLS. These county hospital laboratories are divided into 6 levels where level-2 offers basic diagnostic services such as rapid tests and blood chemistry whereas level 5 is a referral hospital operating with the highest laboratory diagnostic capacity within a county and level 6 are national reference laboratories (e.g., NPHLS). Laboratory mapping of level 2–6 laboratories allowed for systematic determination of the most effective use of laboratory capacity in the country and specimen referral systems as well as identification of the existing gaps. In collaboration with NPHLS and the Association of Public Health Laboratories (APHL), we developed a protocol to map all level laboratories in Kenya. There were three phases of this project starting in 2015. In the first pilot phase, we developed the tools and procedures to collect accurate data pertaining to the capacity of 30 laboratories located in three counties (Kiambu, Kisumu and Laikipia) within the NPHLS laboratory network. This information served to guide the second phase of this project, which consisted of estimating the resources required for data collection in the 47 counties and the frequency of sampling to keep the information for each laboratory updated to allow for the referral network to be developed and maintain updated. In the third phase, we then mapped 1776 tier level 2–6 laboratories within 40 counties in Kenya. Mapping was delayed for the remaining 7 counties due to security issues. We determined the diagnostic capacity of these laboratories using a standardized questionnaire, and geomapped their location. The aim is to use this information to develop a referral and reporting system within the country to rapidly refer test results and specimen transport within the counties or to NPHLS for surveillance and disease outbreak investigations. The referral system was designed to refer specimens from lower to higher level by diagnostic capacity of the 12 Kenya designated priority diseases, 8 zoonotic diseases and 18 especially dangerous pathogens and contaminants. For example, if Machakos county was unable to diagnose a suspected cholera case at a level 3 laboratory, those mapping data would identify the closest level tier 5 county hospital laboratory with cholera culturing capacity for referral of the specimen. If within that county the diagnostic capacity did not exist, those mapping data would provide the closest neighboring county with cholera diagnostic capacity or the specimen would be referred to NPHLS.

Laboratory Information Systems (LIS) ties these GHSA laboratory capacity building activities by supporting the real-time reporting of infectious diseases required by Integrated Disease Surveillance and Response (IDSR) and the IHR. Using LIS platforms (Labware and Basic Laboratory Information System (BLIS)) established by DGHT and our implementing partners ITECH, we assessed the expansion of LIS to report other pathogens in addition to HIV and TB in two counties in Kenya, across a total of 26 facilities (13 facilities per county). A questionnaire was administered to collect information from county and sub county surveillance officers as well as the facility focal persons for Information and Communication Technology, surveillance and laboratory. The goal of the project is to support eventual increased efficiency, sensitivity and timeliness of IDSR reporting through establishment of automated LIS. Although HIV and TB reporting was electronic, our assessment of the 26 selected facilities for other pathogens revealed that collation of surveillance data at the facilities was manual and paper-based while transmission of these data to sub county surveillance officers was through an unstructured SMS which is then fed to the web-based IDSR website. Currently, we are coordinating the strengthening of LIS in the two pilot sites for AMR, Thika and Kitale counties, which had a functional LIS supported by CDC DGHT and EAPHLS (World Bank) respectively, but only for HIV data. DGHP supported the creation of a microbiology module to allow for reporting microbiology surveillance laboratory data to a central repository at NPHLS. Future plans are to continue to expand the LIS to other facilities with proprietary systems and then progress to lower tiered facilities using BLIS, an open source software.

Lastly, DLSP provides laboratory capacity by mentoring interns and sponsoring training courses in coordination with pathogen specific subject matter experts from CDC. Over the last decade DLSP trained technical staff from Kenya and neighboring countries in PCR, ELISA and microbiology diagnostic techniques to detect multiple pathogens (Fig. 2). In addition, 6–10 interns from various local universities are accepted annually to train from 6 months to 1 year. These students then move on to other local or regional institutes to continue their careers as scientists and strengthen this capacity in Kenya and beyond.

### Biosecurity and biosafety

Kenya has a biosafety program developed by DGHT and MoH which established containment practices of those individuals working in the laboratory and principles to avoid unintentional exposure to pathogens or biologicals however this program did not include biosecurity which is the physical containment of a pathogen to protect both animals and humans from either intentional exposure to harmful biological agents [38]. The biosecurity component was included in the Sandia-DTRA developed curriculum primarily targeting biosafety professionals under Kenya’s Veterinary Services. Thus DLSP in conjunction with multiple partners will support and assist with the development of a new biosecurity and biosafety curriculum and training program as defined in the recently adapted (2017) national strategic plan for MoH NPHLS. To build on the MoH existing biosafety curriculum developed with support of DGHT and the financial support of the World Bank, DSLP with the support of DTRA will add the biosecurity portion to this platform and support the merge of these separate curricula to develop and implement a single overarching Kenyan national biosafety and biosecurity curriculum. This curriculum will include five main modules: 1) laboratory quality management systems for safety, 2) biohazard and chemical waste management, transport and tracking of biohazard material, 3) biorisk assessments, mitigation, and reporting 4) occupational safety and infection prevention and control and 5) safety equipment, facility design (biocontainment) and emergency response program. In support of this program, DGHP will support the training of laboratory staff on the use of personal protective equipment, safe transport and tracking of biohazard specimens, biohazard waste management and disposal and good laboratory practice for specimen management for diagnostic testing.

## Conclusion

Kenya’s laboratory expertise and capacity has expanded since 2005 to include the detection of a wide range of pathogens that are threats to global public health. DGHP Kenya has supported KEMRI by establishing DLSP to support this effort and leveraged established platforms from all CDC programs in Kenya such as HIV, TB and malaria in strengthening national laboratory capacity especially at the county level. Moreover, DLSP has participated in over 50 research projects which have yielded over 180 publications. These studies have increased the diagnostic testing capacity of the CDC supported KEMRI and MoH laboratories and thereby contributed to a more rapid detection and control of public health threats thus enhanced global health security. Also, the expertise of DLSP has been harnessed through participation in disease outbreak investigations in Kenya and surrounding countries with measurable public health impact. As a result of these efforts, DGHP-Kenya has supported the establishment of > 100 assays for 153 pathogens for surveillance, disease outbreak investigations and research in Kenya, provided reference laboratory support for the investigations of 205 outbreaks from 2007 to 2016, and detected 2 pathogens not previously diagnosed in Kenya. DLSP support to the national-level laboratories has led to WHO accreditation for the national microbiology laboratory and by 2018 the Kenya NIC is expected to be accredited by the WHO.

In recent years as part of GHSA, DLSP through NPHLS, has transferred laboratory capacity country-wide to various tier level laboratories in strategic locations informed by our GHSA-sponsored laboratory mapping project and through expansion of surveillance for AMR, respiratory disease and AFI, to ultimately provide a network of decentralized laboratories and high quality reference laboratories prepared to respond rapidly to and prevent nascent public health events and threats to Kenya and worldwide. This capacity was recently recognized by the newly established African Centers of Disease Control which has designated Kenya as a regional collaborating center, to support disease prevention and outbreak response throughout the region.
